# Identification, structure, and characterization of an exopolysaccharide produced by *Histophilus somni *during biofilm formation

**DOI:** 10.1186/1471-2180-11-186

**Published:** 2011-08-19

**Authors:** Indra Sandal, Thomas J Inzana, Antonio Molinaro, Christina De Castro, Jian Q Shao, Michael A Apicella, Andrew D Cox, Frank St Michael, Gretchen Berg

**Affiliations:** 1 Center for Molecular Medicine and Infectious Diseases, Virginia-Maryland Regional College of Veterinary Medicine, Virginia Tech, Blacksburg, Virginia, USA; 2 Department of Organic Chemistry and Biochemistry University of Naples "Federico II", Italy; 3 Department of Microbiology, University of Iowa, Iowa City, IA, USA; 4 National Research Council, Ontario, Canada; 5 Division of Pulmonary Medicine, University of Utah School of Medicine, Salt Lake City, UT, USA; 6 Department of Medicine, Boston University School of Medicine, Boston, MA

## Abstract

**Background:**

*Histophilus somni*, a gram-negative coccobacillus, is an obligate inhabitant of bovine and ovine mucosal surfaces, and an opportunistic pathogen responsible for respiratory disease and other systemic infections in cattle and sheep. Capsules are important virulence factors for many pathogenic bacteria, but a capsule has not been identified on *H. somni*. However, *H. somni *does form a biofilm *in vitro *and *in vivo*, and the biofilm matrix of most bacteria consists of a polysaccharide.

**Results:**

Following incubation of *H. somni *under growth-restricting stress conditions, such as during anaerobiosis, stationary phase, or in hypertonic salt, a polysaccharide could be isolated from washed cells or culture supernatant. The polysaccharide was present in large amounts in broth culture sediment after *H. somni *was grown under low oxygen tension for 4-5 days (conditions favorable to biofilm formation), but not from planktonic cells during log phase growth. Immuno-transmission electron microscopy showed that the polysaccharide was not closely associated with the cell surface, and was of heterogeneous high molecular size by gel electrophoresis, indicating it was an exopolysaccharide (EPS). The EPS was a branched mannose polymer containing some galactose, as determined by structural analysis. The mannose-specific *Moringa M *lectin and antibodies to the EPS bound to the biofilm matrix, demonstrating that the EPS was a component of the biofilm. The addition of *N*-acetylneuraminic acid to the growth medium resulted in sialylation of the EPS, and increased biofilm formation. Real-time quantitative reverse transcription-polymerase chain reaction analyses indicated that genes previously identified in a putative polysaccharide locus were upregulated when the bacteria were grown under conditions favorable to a biofilm, compared to planktonic cells.

**Conclusions:**

*H. somni *is capable of producing a branching, mannose-galactose EPS polymer under growth conditions favorable to the biofilm phase of growth, and the EPS is a component of the biofilm matrix. The EPS can be sialylated in strains with sialyltransferase activity, resulting in enhanced density of the biofilm, and suggesting that EPS and biofilm formation may be important to persistence in the bovine host. The EPS may be critical to virulence if the biofilm state is required for *H. somni *to persist in systemic sites.

## Background

*Histophilus somni *(*Haemophilus somnus*) is a host-specific, gram-negative coccobacillus, and an opportunistic pathogen of cattle and sometimes sheep that is responsible for a variety of systemic infections, including meningoencephalitis, pneumonia, myocarditis, septicemia, and reproductive failure [1,2]. Hallmarks of *H. somni *infection include septicemia, by which the organism can disseminate to various tissues such as the brain, heart, and joints [1-3], and adherence to and inflammation of vascular endothelial cells [4,5]. Pathogenic isolates of *H. somni *share many virulence attributes with human-specific mucosal pathogens that are designed to resist host defense mechanisms. For example, the structure of the lipooligosaccharide (LOS) of *H. somni *is remarkably similar to that of *Neisseria gonorrhoeae*, including an outer core that mimics the structure of lacto-*N*-neotetraose on the glycosphingolipid of mammalian cells [6-8]. Furthermore, like *Haemophilus influenzae*, the *H. somni *LOS outer core undergoes a high rate of phase variation due to variable number tandem repeats in the genes that encode for the LOS glycosyl transferases [9,10]; the LOS is also decorated with *N*-acetylneuraminic acid (Neu5Ac or sialic acid) and phosphorylcholine, which can contribute to resistance to host defenses and adaptation to specific host sites [11,12]. Other *H. somni *virulence attributes include immunoglobulin binding proteins [13,14], cell adhesions [3], resistance to the bactericidal activity of serum [15], survival in and inhibition of the oxidative burst of phagocytic cells [16-19], toxicity to epithelial cells [20,21], and induction of apoptosis of endothelial cells [22-24]. However, a few strains that have been isolated from the genital tract are serum-sensitive and less virulent or avirulent [15]. Determination of the genome sequence of *H. somni *avirulent strain 129Pt from the healthy bovine prepuce [25], and 2336 from bovine pneumonia (sequence accession number NC_010519) revealed many genetic deletions and insertions that may be associated with differences in the virulence of these two strains.

Many species in the family *Pasteurellaceae *are encapsulated, including *Haemophilus influenzae, H. parasuis, Actinobacillus pleuropneumoniae, Mannheimia haemolytica*, and *Pasteurella multocida*. However, *H. somni *has been reported to be nonencapsulated, based on ruthenium red staining and electron microscopy [1,26,27]. Nonetheless, Miller et al. [28] reported the presence of a polysaccharide other than LOS in *H. somni *cultures, although the composition and relationship of this polysaccharide to *H. somni *was not determined. The capability of *H. somni *to produce a biofilm under growth conditions that favor low oxygen tension and low shear forces has been described [29], but the composition of the matrix making up the biofilm is not yet well characterized. In most bacteria the biofilm matrices normally consist largely of polysaccharide [30].

A comparative analysis of extracts from cells grown anaerobically and in a candle extinction jar revealed the presence of a polysaccharide in anaerobic extracts only. Subsequently it was determined that the polysaccharide could be efficiently purified from broth cultures grown to late stationary phase under low aeration conditions favorable to biofilm formation [29]. We have determined that this high molecular weight polysaccharide from *H. somni *is a branched mannose polymer, and a component of the *H. somni *biofilm. Following genome sequencing of 129Pt and 2336, putative genes that may be responsible for production of this polysaccharide were identified [[25], Siddaramappa S CJ, Duncan AJ, Gillaspy AF, Carson M, Gipson J, Gipson M, Orvis J, Zaitshik J, Barnes G, Brettin TS, Bruce D, Chertkov O, Detter JC, Han CS, Tapia R, Thompson LS, Dyer DW, Inzana TJ: Genome sequence of *Histophilus somni *strain 2336 from bovine pneumonia and comparison to commensal strain 129Pt reveals extensive horizontal gene transfer and evolution of pathogenesis. Submitted]. Most of these genes were found to be upregulated under conditions that favor biofilm formation.

## Methods

### Bacterial strains and growth conditions

*H. somni *2336 is a pathogenic isolate from bovine pneumonia, 738 is an LOS phase variant of 2336 obtained following subculture and passage in a bovine, and 129Pt is a non-pathogenic commensal from the healthy bovine prepuce [15]. The bacteria were grown on Columbia agar with 5% sheep blood (CBA) in 3-5% CO_2_, in Columbia broth, or Terrific broth (Difco, BD Diagnostic Systems, Sparks, MD); the latter two supplemented with 0.1% Trizma base (no pH adjustment), 0.01% thiamine monophosphate (TMP) [31] (CTT or TTT, respectively), and 1% glucose. The bacteria were grown at 37°C or 42°C with rapid shaking (~200 rpm) in flasks with a large headspace and harvested in early stationary phase (~5 × 10^9 ^colony forming units [CFU]/ml). Alternatively, the bacteria were grown under low oxygen tension in a bottle filled with medium to minimize the headspace and shaken slowly (75 rpm) to favor biofilm formation [29]. Bacteria were also grown in a strict anaerobic environment on CBA in a BD GasPak system (BD Diagnostic Systems), or in CTT containing Oxyrase for Broth™ (Oxyrase, Mansfield, OH). For some experiments, the medium was supplemented with 2% NaCl, or the bacteria were harvested during mid- to late-stationary phase (48-72 h post-inoculation). For growth supplementation with Neu5Ac, 1 mg (50-μg/ml final concentration) of Neu5Ac (Sigma Chemical Co.) was added to CBA, TTT, or to a chemically defined medium [31].

### Polysaccharide purification

*H. somni *was grown on CBA plates incubated in 5% CO_2 _or anaerobic conditions for 48-72 h at 37°C. The cells were scraped from the plates and suspended in phosphate buffered saline, pH 7.2, (PBS) to a turbidity of 150 Klett units (about 10^9 ^CFU/ml). After vigorous vortexing at room temperature, the cell suspension was incubated at 37°C for 1 h, vortexed again, and the cells removed by centrifugation (10,000 × *g *for 15 min). Cetavlon (hexadecyltrimethyl ammonium bromide) was added to a final concentration of 0.005 M. Any precipitate that formed was harvested and solubilized in distilled water. No further purification was done on this sample. Alternatively, the bacteria were grown to late stationary phase in CTT (48-72 h post-inoculation), the bacteria harvested as above, and Cetavlon added to the supernatant. Any precipitate that formed following addition of Cetavlon was further purified by enzyme digestion (RNase, DNase, and Proteinase K), phenol extraction, and ultracentrifugation to remove LOS, as described for purification of the capsular polysaccharide of *Actinobacillus pleuropneumoniae *[32].

The bacteria were also grown at 37°C in filled 1-L bottles containing TTT with shaking at 75 rpm for 4-5 days. The clear supernatant was carefully removed and the sediment was extracted with 45% aqueous phenol at room temperature, digested with DNase, RNase, and Proteinase K, and subjected to ultracentrifugation at 125,000 × *g *at 4°C, as described for purification of *H. somni *LOS [33], except that the supernatant from the ultracentrifugation step was retained. Polysaccharide in the supernatant was precipitated by the addition of 30 mM sodium acetate (final) and 5 volumes of cold (-20°C) 95% ethanol, and incubated at -20°C for at least 4 hours. The pellet obtained by centrifugation was suspended in distilled water, and eluted through a Sephacryl S-400 column (2.5 × 50 cm) with distilled water as eluent. The first fractions containing carbohydrate (determined by phenol-sulfuric acid assay) [34] were pooled and lyophilized.

For comparative analysis of polysaccharide production under various environmental conditions, the bacteria were cultured in CTT at 37°C either anaerobically (with Oxyrase) or with 2% NaCl aerobically, or at 42°C aerobically. All cultures were grown to 4 × 10^9 ^CFU/ml (early stationary phase). The bacteria were harvested and 0.005 M Cetavlon (final concentration) was added to the supernatants to precipitate large molecular mass, negatively charged components. The precipitate was then solubilized with 0.9 M NaCl, 5 volumes of cold ethanol were added, and the mixture incubated at -20°C overnight. The precipitate was resuspended in water, lyophilized, and weighed to determine the amount of polysaccharide in each sample. The cell pellets were washed with PBS and the concentration of protein in each sample was determined by BCA protein assay (Pierce, Rockford, IL).

### Polyacrylamide gel electrophoresis and alcian blue silver staining

Polyacrylamide gel electrophoresis (PAGE) for polysaccharides was done as described by Pelkonen et al. [35], followed by alcian blue and silver staining by a modified method of Min and Cowman [36] using a Bio-Rad silver stain kit.

### Immune serum

Rabbits were immunized subcutaneously in 4 different sites with a total of 50 μg of purified polysaccharide (in 1 ml of sterile water) mixed 1:1 with Freund's Complete Adjuvant, followed by a second immunization 3 weeks later with the same formulation of 50 μg of polysaccharide in Freund's Incomplete Adjuvant. The rabbits were then immunized intravenously with 50 μg of the polysaccharide until high-titer immune serum was obtained [37]. The IgG fraction of the antiserum was isolated by Protein A/G affinity chromatography [38].

### Immuno-transmission electron microscopy (ITEM) for analysis of polysaccharide on cells and in the biofilm

To determine if the polysaccharide formed a well-associated structure around cells of *H. somni*, the bacteria were grown anaerobically or in CO_2_, and gently scraped off plates to a turbidity of 150 Klett units (~10^9 ^cells/ml). Immunofixation was done as previously described [39] using 1.5 ml of bacterial suspension incubated for 1 h at 37°C with 1 ml of a rabbit IgG (0.3 mg/ml) to the polysaccharide. Thin sections were examined with a JEOL 100 CX-II transmission electron microscope.

Biofilms were grown on coverslips in TTT to stationary phase [40], and fixed overnight in a 1-ml mixture of 4% paraformaldehyde and 5% dimethyl sulfoxide. Samples were then embedded *in situ *in OCT (Sakura Finetek USA, Inc., Torrance, Calif.) on the coverslip surface upon which they were formed. For cryo-ITEM the coverslip was removed by freezing the sample in liquid nitrogen and shattering the glass, leaving the biofilm within the OCT. The OCT block was cut into 10 μm thick sections using a Cryostat (MICROM HM 505E) [41].

OCT sections were washed with PBS, blocked with 5% NGS (normal goat serum) (Electron Microscopy Sciences, Hatfield, PA) for 15 min, and washed with PBS. The sections were incubated with a 1:25 dilution of polyclonal rabbit antiserum to the polysaccharide at 4°C overnight, washed with PBS, and incubated with the secondary antibody (goat anti-rabbit IgG) conjugated to 10-nm colloidal gold (Electron Microscopy Sciences) for 2 hours. The samples were subsequently washed in PBS, fixed in 2.5% glutaraldehyde in 0.1 M sodium cacodylate buffer, treated with 1% osmium tetroxide, dehydrated through a graded series of ethanol (30%, 50%, 70%), and flat-embedded in Spurr's resin using a Chien embedding mold (Polysciences, Niles, IL). Thin sections (70 nm) were cut with a Leica EM UC-6E ultramicrotome, collected on Formvar-coated nickel grids, and stained with uranyl acetate and lead citrate. The grids were dried and observed using a JEOL 1230 TEM.

### Scanning electron microscopy (SEM)

*H. somni *2336 and 129pt were grown as a biofilm in chemically defined medium [31] with and without Neu5Ac (50 μg/ml) on glass coverslips in a 12-well plate (Falcon 3911, Microtest), and incubated for 5 days at 37°C without shaking. The coverslips were washed gently with PBS and fixed in 2.5% glutaraldehyde. The samples were processed as described [40], and examined using a Philips 505 scanning electron microscope.

### Lectin binding to biofilms

The OCT resin sections were incubated with the fluorescein-conjugated *Moringa M *lectin (EY Laboratories, San Mateo, Calif.), which is specific for mannose, and counter-stained with the nucleic acid stain To-Pro3 (Molecular Probes, Invitrogen) as described [41]. The sections were washed in PBS three times, mounted with a coverslip, and examined by confocal laser scanning microscopy with red and green channels.

### Analytical and structural methods

To determine if supplementation of cultures with Neu5Ac modified LOS under different culture conditions, LOS was extracted from bacteria grown as a biofilm, as planktonic cells, or on blood agar plates supplemented with and without Neu5Ac as previously described, and then O-deacylated (OdA LOS) (12). The OdA LOS samples were analyzed by negative ion electrospray mass spectrometry (ES-MS) on a VG Quattro triple quadrupole mass spectrometer (Fisons Instruments) with selective ion scanning at *m*/*z *290, specific for Neu5Ac, as described previously [12].

To determine the presence of Neu5Ac on the polysaccharide from cells grown as a biofilm, polysaccharide purified from the biofilm (1 mg) was dried over P_2_O_5 _for 1 h under diminished pressure and treated with methanol/2 M HCl at 80°C for 18 h. The solution was extracted twice with equal volumes of *n*-hexane to remove contaminant fatty acid methyl esters, the methanolic phase was dried, and the *O*-methyl glycosides were acetylated with dry pyridine (200 μl) and Ac_2_O (100 μl) at 80°C for 30 min. The reactants were removed by evaporation, and the mixture of peracetylated *O-*methyl glycosides was analyzed by gas-liquid chromatography-mass spectrometry (GLC-MS).

Sugar residues and their absolute configuration were determined by GLC and GLC-MS, which were carried out as described [8]. Monosaccharides were identified as acetylated *O*-methyl glycoside derivatives. After methanolysis (2 M HCl/MeOH, 85°C, 24 h) and acetylation with acetic anhydride in pyridine (85°C, 30 min) the polysaccharide sample was analyzed by GLC-MS. Linkage analysis was carried out by methylation, as described [42]. The sample was hydrolyzed with 4 M trifluoroacetic acid (100°C, 4 h), carbonyl-reduced with NaBD_4_, acetylated, and analyzed by GLC-MS.

For enzymatic hydrolysis of the polysaccharide, 10 mg was dissolved in 50 mM Na^+^CH_3_COO^- ^(2 ml) and treated with α-mannosidase (200 μl, Sigma) at 30°C for 7 days. After lyophilization the sample was fractionated through a 1.5 × 100 cm column of Sephadex G-15 (Pharmacia), and eluted with 10 mM NH_4_HCO_3 _at a flow rate of 45 mL/h. Fraction volumes of 2 ml were collected.

Acetolysis of mannan (30 mg) was performed as reported [43]. The acetylated products were applied to a column (1 × 150 cm) of TSK-40, and eluted with distilled water at a flow rate of 14 ml/h at room temperature; 2.5 ml fractions were collected. The fractionation yielded four fractions, as described in results.

Nuclear magnetic resonance (NMR) spectroscopy was used to obtain structural details of the polysaccharide. For structural assignments, 1D and 2D ^1^H-NMR spectra were recorded from a solution of 2 mg of polysaccharide in 0.5 ml of D_2_O, at 300 K, at pD 7, using a Bruker 600 DRX equipped with a cryo probe. The spectra were calibrated with internal acetone [δ_H _2.225, δ_C _31.45]. ^31^P NMR experiments were carried out using a Bruker DRX-400 spectrometer, with aqueous 85% phosphoric acid used as an external reference (0.00 ppm). Rotating frame Overhauser enhancement spectroscopy (ROESY) data sets (t_1 _× t_2_) were measured using 4096 × 256 points with a mixing time of 200 ms. Double quantum-filtered phase-sensitive correlation spectroscopy (COSY) experiments were performed with 0.258 s acquisition time, using data sets of 4096 × 256 points. Total correlation spectroscopy experiments (TOCSY) were performed with a spinlock time of 100 ms, using data sets (t_1 _× t_2_) of 4096 × 256 points. In all homonuclear experiments the data matrix was zero-filled in the *F*1 dimension to give a matrix of 4096 × 2048 points, and was resolution-enhanced in both dimensions by a sine-bell function before Fourier transformation. Coupling constants were determined on a first order basis from 2D phase-sensitive double quantum filtered correlation spectroscopy (DQF-COSY) [44]. Heteronuclear single quantum coherence (HSQC) and heteronuclear multiple bond correlation (HMBC) experiments were measured in the ^1^H-detected mode via single quantum coherence with proton decoupling in the ^13^C domain, using data sets of 2048 × 256 points. Experiments were carried out in the phase-sensitive mode. A 60 ms delay was used for the evolution of long-range connectivities in the HMBC experiment. In all heteronuclear experiments the data matrix was extended to 2048 × 1024 points using forward linear prediction extrapolation.

### Real-time quantitative reverse transcription PCR (qRT-PCR)

Extraction of total RNA was performed from 3, 5, and 7 day-old biofilms using Total RNA Isolation (TRI) reagent (Molecular Research Centre, Inc., Cincinnati, OH) [45]. Biofilms were grown in 1 L of broth as described above. The clear supernatant was carefully removed and the biofilm at the bottom of the flask was treated directly with TRI reagent following the manufacturer's protocol. To remove contaminating genomic DNA, approximately 10 μg of RNA was treated using Qiagen's RNeasy on-column DNase I (*Q*, 2.7 U DNase I/10 μg RNA), followed by Qiagen RNeasy MinElute (for DNase I removal) according to the manufacturer's protocol. The RNA concentration was determined spectrophotometrically using a Nanodrop ND-1000 instrument (Nanodrop Technologies, Wilmington, DE), and the integrity of the RNA was assessed by agarose gel electrophoresis. Planktonic cells were collected after centrifugation (6000 × *g *at 4°C) and resuspended in TRI reagent for extraction of RNA. Cell pellets were stored at -80°C until needed for RNA isolation.

Amplification, detection, and analysis of mRNA was performed using the ABI-Prism 7000 sequence detection system with a SYBR Green PCR master mix (Applied Biosystems, Carsbad, CA). The corresponding oligonucleotide primers were designed using Primer Express software (Applied Biosystems) and optimized for uniform size (90-100 bp) and consistent melting temperature (55°C). For each set of primers, a standard amplification curve was plotted [critical threshold cycle (C_t_) against log of concentration] and only those with a slope of approximately -3 were considered reliable primers. SuperScript III First-Strand Synthesis System for qRT-PCR (Invitrogen; C The qRT-PCR reaction mixture contained 1× SYBR Green PCR master mix (Applied Biosystems), 1 μl cDNA, and 0.5 μM of the forward and reverse PCR primers. Initial denaturation was at 95°C for 10 min, followed by a 40-cycle amplification of denaturation at 95°C for 15 s, and annealing and extension at 60°C for 1 min. The critical threshold cycle, C_t_, was defined as the cycle in which fluorescence becomes detectable above the background fluorescence, and is inversely proportional to the logarithm of the initial number of template molecules. A standard curve was plotted for each primer set with C_t _values obtained from amplification of known quantities of *H. somni *cDNA. The standard curves were used for converting the C_t _values into the relative number of cDNA molecules. Control reactions were used to determine any contamination by genomic DNA. The levels of expression of all genes tested by qRT-PCR were normalized using the housekeeping gene tryptophanyl-tRNA synthetase (*trpS*) of *H. somni *as an internal standard. There was no significant difference in the expression of the *trpS *under the different conditions or in the various samples tested (data not shown). Each assay was performed with three independent RNA samples in triplicate.

### Statistical analysis

The Student's *t *test was used to calculate the statistical differences between the mean levels of polysaccharide expression of experimental samples (biofilm grown cells) and control samples (planktonic cells). A *P *value < 0.05 was considered significant. All statistical analyses were done using InStat software (InStat, San Diego, CA).

## Results

### Identification of a novel *H. somni *surface component produced during anaerobic growth

**Figure 1:**
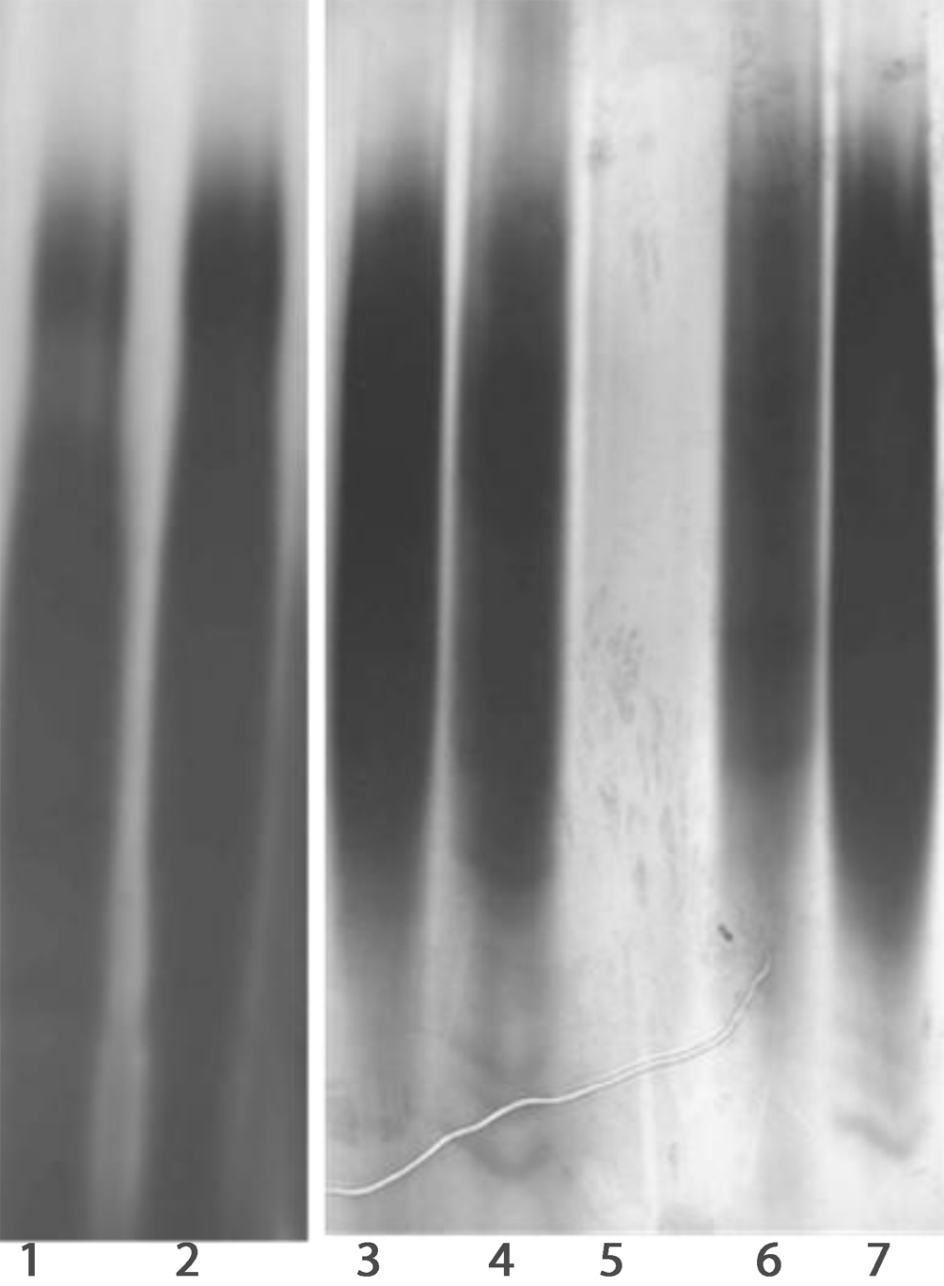
**Electrophoretic profiles of semi-purified Cetavlon precipitates and biofilm**. Bacteria were grown anaerobically on plates or to late stationary phase, Cetavlon added, and precipitates extracted, as described in Methods. Each extract was loaded onto 25% polyacrylamide gels, followed by electrophoresis and staining with Alcian blue and silver. Lanes: 1 and 2, 20 μg and 30 μg of EPS extracted under growth conditions favorable to biofilm formation; 3 and 4, 20 μg and 30 μg of EPS extracted from cells grown to late stationary phase in broth, respectively; 5, buffer alone; 6 and 7, 20 μg and 30 μg of EPS extracted from cells grown anaerobically on plates, respectively.

To determine if there was variation in expression of membrane components under different environmental conditions, *H. somni *738 was grown on CBA plates in 3-5% CO_2 _or under anaerobic conditions for 48 h at 37°C. The bacteria were harvested from the plates as described in methods, and Cetavlon was added to the supernatant (0.005 M, final concentration); LOS and protein-enriched outer membranes were prepared from the cell pellets [46,47]. No substantial qualitative differences were detected in the electrophoretic profiles of the LOS or membrane proteins of bacteria grown on CBA under CO_2 _or anaerobic conditions (data not shown), although growth of *H. somni *under anaerobic conditions was poor. Nonetheless, when Cetavlon was added to the supernatant of cells washed off CBA plates incubated under anaerobic conditions, a large precipitate formed, whereas little or no precipitate formed from the supernatant of cells grown on CBA in CO_2 _(data not shown). The Cetavlon precipitate was solubilized in distilled water, and greater than 90% of the precipitate was determined to be carbohydrate. However, it was not LOS, as determined by polyacrylamide gel electrophoresis and silver staining for LOS (data not shown). Electrophoresis of the Cetavlon precipitate followed by staining with alcian blue and ammoniacal silver demonstrated a heterogeneous profile, typical of high molecular size polysaccharide (Figure 1).

### Immuno-transmission electron microscopy of *H. somni *grown under anaerobic conditions or CO_2_

**Figure 2:**
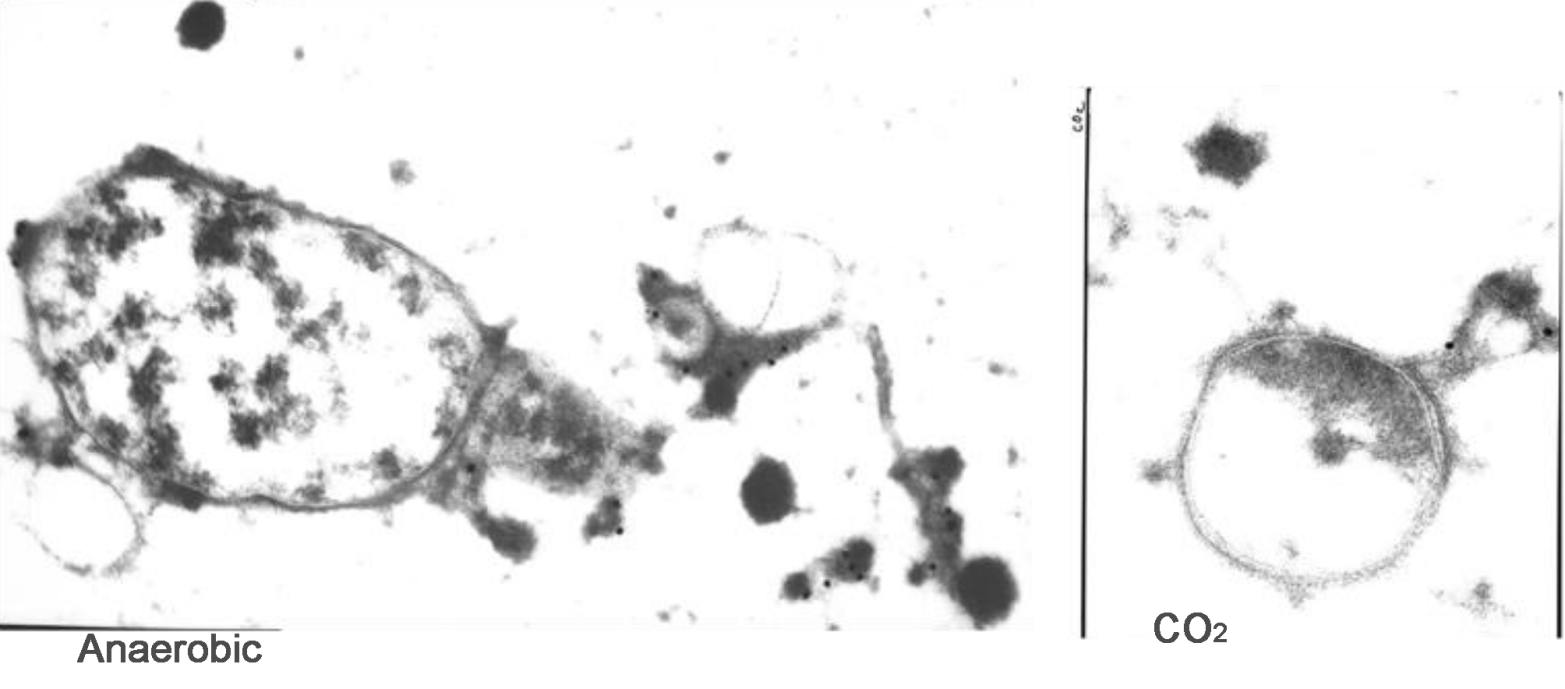
**Immuno-transmission electron microscopy**. Affinity-purified IgG was prepared from antiserum to isolated EPS made in rabbits, and incubated with whole cells that were gently scraped off plates, followed by Protein-A gold particles. The dark particles binding to the extracellular matrix (arrows) are Protein A-gold particles binding to immunoglobulins. Note that none of the Protein A-gold particles bound to the cell membrane, but were bound to extracellular material shed from the cell. More of this extracellular material was present when cells were grown anaerobically (left) than when cells were grown in CO_2 _(right).

The polysaccharide from Cetavlon precipitates obtained from scaled up anaerobic cultures was further purified, as described in methods, and used to immunize a rabbit. The IgG from this antiserum was incubated with cells gently scraped off agar plates incubated in an anaerobic or 3-5% CO_2 _atmosphere, and processed for ITEM. The Protein-A gold particles clearly bound to material that was shed from the cell surface and in relatively large quantities (Figure 2), indicating it was an exopolysaccharide (EPS). However, little of this material was produced by bacteria incubated in CO_2 _(Figure 2). Cells incubated with nonspecific IgG did not bind Protein-A gold particles (not shown).

### Effect of growth conditions on *H. somni *exopolysaccharide production

EPS production by strain 2336 appeared to be enhanced under stress or growth conditions that did not favor rapid or abundant growth. Therefore, to determine the relative amount of EPS produced per cell, the purified EPS content (dry weight) was determined in relation to the total amount of protein in the sample (Table 1). EPS production appeared to be upregulated in late stationary phase, relative to exponential phase growth at 37°C. In addition, the amount of EPS/cellular protein was further enhanced when the bacteria were grown to the same density at early stationary phase under anaerobic and high salt conditions, but not at 42°C.

**Table 1:** *H.somni *EPS production under various growth conditions in relation to cellular protein content

Growth Conditions	Relative Amount of EPS (mg EPS/mg protein)
37°C (stationary phase)	50.7
42°C (log phase)	25.5
37°C (anaerobic growth)	69.2
37°C (supplementation with 2% NaCl)	95.1

### *H. somni *exopolysaccharide production

As mentioned above, changing the environmental conditions to enhance *H. somni *EPS production, such as anaerobic conditions, often resulted in poor bacterial growth, making it difficult to purify large amounts of EPS. Although very little EPS was produced in broth during log phase, more EPS was produced after the bacteria reached late stationary phase. Therefore, the bacteria were grown in CTT for 48-72 h prior to harvesting the bacteria, enabling the EPS to be purified from the culture supernatant (Figure 1).

Larger quantities of EPS could be isolated by incubating the bacteria in 1 L of TTT in a 1 L bottle incubated at 37°C and rotated slowly at 70 rpm. After about 24 h incubation the medium was uniformly turbid with planktonic bacteria, but after 48-72 h incubation a large biofilm-like mass became established on the bottom of the flask. The top 900 ml of clear medium was removed and the EPS was purified from the sediment.

### Structural analysis of the exopolysaccharide

**Figure 3:**
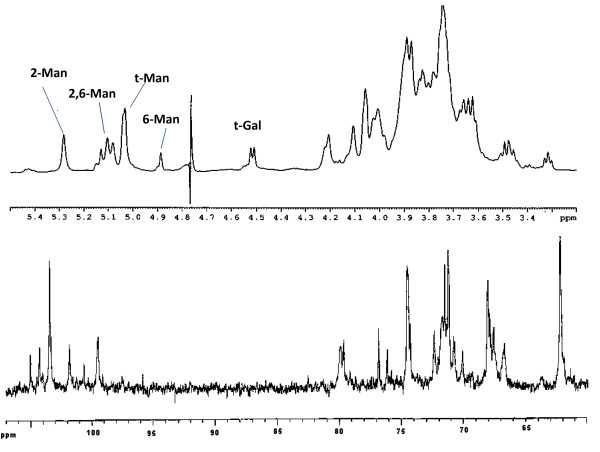
**The ^1^H- (A) and the ^13^C-NMR spectra from the purified EPS of *H. somni *2336**. The spectrum was recorded in D_2_O at 25°C, relative to the HOD signal at 4.78 ppm.

Chemical analyses carried out by GLC-MS of the acetylated *O*-methyl glycoside and *O*-oct-2-yl glycoside derivatives yielded two monosaccharides, both in D configuration: mannose and traces of galactose. Methylation analysis showed the presence of derivatives of terminal Gal*p*, terminal Man*p*, 2-substituted Man*p*, 3-substituted Man*p*, 6-substituted Man*p*, and 2,6-substituted Man*p*. On the basis of chemical data it could be hypothesised that the structure consisted of a mannan backbone to which other mannose (and some galactose) branching residues were attached. The ^1^H-NMR and ^13^C NMR spectra appeared rather complex (Figure 3).

Chemical shifts were assigned utilizing DQF-COSY, TOCSY, ROESY, HSQC, and HMBC experiments (Table 2). Anomeric configurations were assigned on the basis of the chemical shifts of the ^3^*J*_H-1, H-2 _values, which were determined from the DQF-COSY experiment, and from the shifts of ^1^*J*_C-1, H-1 _values derived from a coupled ^1^H,^13^C-HSQC. Based on the TOCSY spectrum from the H-2 proton signal for all the spin systems, it was possible to assign all of the resonances, and from these, all the ^13^C resonances from the HSQC spectrum.

**Table 2:** ^1^H and ^13^C NMR data of the galactomannan fraction from *Histophilus somni *2336

Residue	1	2	3	4	5	6
2-Man*p*	5.28	4.10	3.91	3.72	3.71	3.87, 3.72
	101.2	79.3	71.0	67.4	75.4	61.8
3-Man*p*	5.16	4.21	3.88	3.65	3.76	3.89, 3.74
	103.2	71.1	79.1	66.0	75.3	62.0
2,6-Man*p*	5.13	4.22	3.87	3.60	3.76	3.88, 3.73
	99.2	79.1	71.1	66.1	74.6	68.0
2,6-Man*p*	5.10	4.03	3.93	3.69	3.80	4.00, 3.70
	99.2	79.6	71.5	67.8	74.6	67.6
t-Man*p*	5.03	4.06	3.86	3.66	3.75	3.89, 3.71
	103.2	71.0	71.2	67.5	76.4	62.1
t-Man*p*	5.04	4.20	3.93	3.62	3.86	3.89, 3.71
	103.2	70.1	70.7	67.9	76.4	62.1
6-Man*p*	4.89	3.98	3.82	3.71	3.88	3.91, 3.73
	100.6	70.6	71.0	67.3	74.8	66.5
t-Gal*p*	4.52	3.32	3.48	3.87	3.84	3.84, 4.21

In the low field anomeric region several signals were present, all identifiable as mannose spin systems (low ^3^*J*_H-1, H-2 _and ^3^*J*_H-2, H-3 _values) experiencing a different magnetic environment. At 5.28 ppm a cluster of signals were present, all indicative of 2-substituted mannose residues. In fact, ^13^C resonance assignments showed the downfield displacement of a C-2 resonance for the spin system, evidently due to glycosylation. Furthermore, at 5.16 ppm a cluster of signals indicated that a 3-substituted mannose was present, as attested by the downfield shift of C-3 resonance at 79.1 ppm. At 5.13 and 5.10 ppm two very similar spin systems were found. Both residues possessed C-2 and C-6 chemical shifts at low fields owing to glycosylation, and were therefore identified as two distinct clusters of 2,6-di-subtituted mannose residues that experienced a slightly different magnetic environment. Likewise, at 5.03 and 5.04 ppm, it was possible to identify two non-substituted mannose residues, as inferred by their ^1^H and ^13^C resonances. The last mannose residue was present at 4.889 ppm and was representative of a 6-substituted mannose, given the downfield shift value of its C-6 resonance. At higher fields (4.52 ppm) another anomeric proton signal was present, which was attributable to the galactopyranose residue present in its β-anomeric configuration (^3^*J*_H-1, H-2 _= 8.1 Hz). Analysis of the TOCSY spectrum made it possible to determine the H-1 to H-4 resonances. In contrast, the H-5 resonance, as in all *galacto*-configured systems, was only visible by NOESY owing to its low coupling constant with H-4, which impaired any transfer of magnetization. The chemical shifts of carbon signals of this latter spin system were taken from HSQC, and indicated there was no glycosylation shift, suggesting the presence of an unsubstituted β-galactopyranose residue.

**Figure 4:**
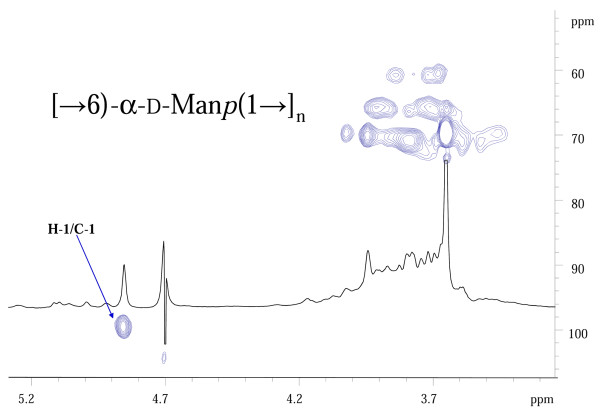
**HSQC and the ^1^H-NMR spectra of the mannosidase-digested polymer that demonstrates the presence of a single abundant peak at 4.89 ppm, which represents the anomeric proton signal of the 6-substituted mannose**.

On the basis of the above chemical and NMR data, and in accordance with reported data [48], it was likely that the EPS was an α-(1→6)-linked, highly branched, comb-like mannopyranan polysaccharide structure with mannopyranose units branched at C-2 with 2-substituted mannose residues. In order to confirm this structural hypothesis, we carried out an enzymatic hydrolysis on 10 mg of the EPS using an *exo*-mannosidase that is able to cleave the branching mannose residues starting from the non-reducing ends. As expected, after purification by gel filtration chromatography, two products were identified. The lower molecular size fraction was mannose (6 mg). The polysaccharide fraction that eluted in the void volume (3 mg) was analysed by NMR spectroscopy, and although still present as part of a heterogeneous polymer, this fraction consisted of only one major residue. The comparison of proton anomeric signal intensities between the polymer and the mannosidase-degraded product showed a remarkable increase in the signal at δ4.89 (6-substituted mannose) with respect to all the other signals (Figure 4). However, it was not possible to observe the galactose signal in this polymer, likely because the amount of galactose in the entire EPS was very low and in the presence of the predominant mannose, disappeared due to background noise. The methylation analysis was in good agreement with this observation, and showed a substantially higher content of 6-substitued mannose. Following the *exo*-mannosidase hydrolyses of the terminal mannose units, it was confirmed that 6-substituted mannose was a constituent of the mannan backbone and that 2- substituted and 3-substituted mannose were present in the oligosaccharide arms.

After establishing the nature of the backbone, an acetolysis reaction was used to determine the identity and length of the branches. This reaction selectively cleaves the (1→6)-glycosidic linkages furnishing, in this case, the intact lateral chains of the polymer with a reducing mannose that was originally present as the 6-substituted residue in the backbone. The crude reaction mixture was separated by TSK-40 gel-filtration chromatography, and yielded four fractions **(1-4) **that were all subjected to a combination of chemical and spectroscopic analyses.

Fraction **1 **was established to be a mannose-reducing tetrasaccharide and contained a slight amount of a tetrasaccharide, in which galactose replaced the non reducing mannose end as follows:

$$\alpha -\text{D}-\text{Man}p-\left(1\to 3\right)-\alpha -\text{D}-\text{Man}p-\left(1\to 2\right)-\alpha -\text{D}-\text{Man}p-\left(1\to 2\right)-\text{D}-\text{Man}p-red.$$

$$\beta -\text{D}-\text{Gal}p-\left(1\to 3\right)-\alpha -\text{D}-\text{Man}p-\left(1\to 2\right)-\alpha -\text{D}-\text{Man}p-\left(1\to 2\right)-\text{D}-\text{Man}-red$$

**Figure 5:**
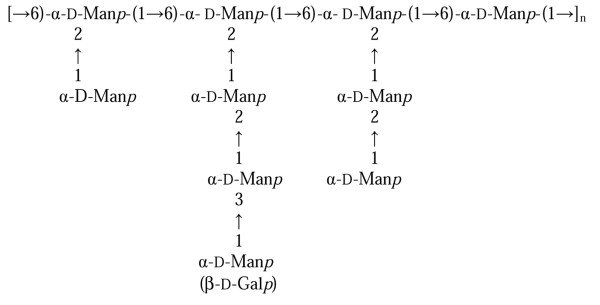
**Proposed structure of the EPS of *H. somni *2336**.

Fraction **2 **was found to be a trisaccharide: α-D-Man*p*-(1→2)-α-D-Man*p*-(1→2)-D-Man-*red*, fraction **3 **consisted of the disaccharide α-D-Man*p*-(1→2)-D-Man-*red*, and fraction **4 **was only composed of reducing mannose. Thus, the acetolysis showed that only three kinds of oligosaccharides were present, which were attached to the main polymer backbone, and that these branches were all attached to O-2 of a 2,6-disubstituted mannose. Moreover, the galactose residue, when present, was only located at the non-reducing end of a tetrasaccharide. Thus, from both selective degradation reactions, it could be concluded that the galacto-mannan polymer is an intricate structure consisting of a 6-substituted mannan backbone with small branching chains (one to three units) of Man*p *residues. Furthermore, the 3-substituted mannose is only present in the trisaccharide lateral chain. The overall structure of this complex EPS is shown in Figure 5.

**Figure 6:**
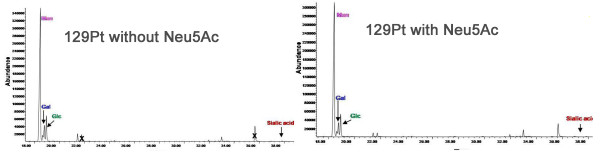
**Chromatogram GC-MS of *H. somni *129 pt grown without Neu5Ac (left) and with Neu5Ac (right)**.

**Figure 7:**
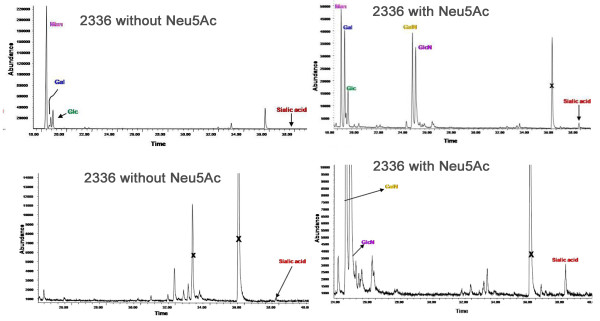
**Chromatogram GC-MS of *H. somni *2336 grown without Neu5Ac (top left) and with Neu5Ac (top right), and chromatogram expansion GC-MS of 2336 grown without Neu5Ac (bottom left) and with Neu5Ac (bottom right)**.

When 2336 and 129Pt were grown with and without Neu5Ac added to the culture medium, only traces of Neu5Ac were present in the purified EPS of 129Pt without Neu5Ac (Figure 6, left panel), with Neu5Ac (Figure 6, right panel), or in 2336 grown without Neu5Ac (Figure 7, left panels). However, a significantly larger quantity of Neu5Ac was present in the EPS of 2336 grown with Neu5Ac (Figure 7, right panels). Furthermore, the EPS also contained two additional aminosugars: *N-*acetylglucosamine and *N-*acetylgalactosamine.

### Association of the exopolysaccharide with biofilm

**Figure 8:**
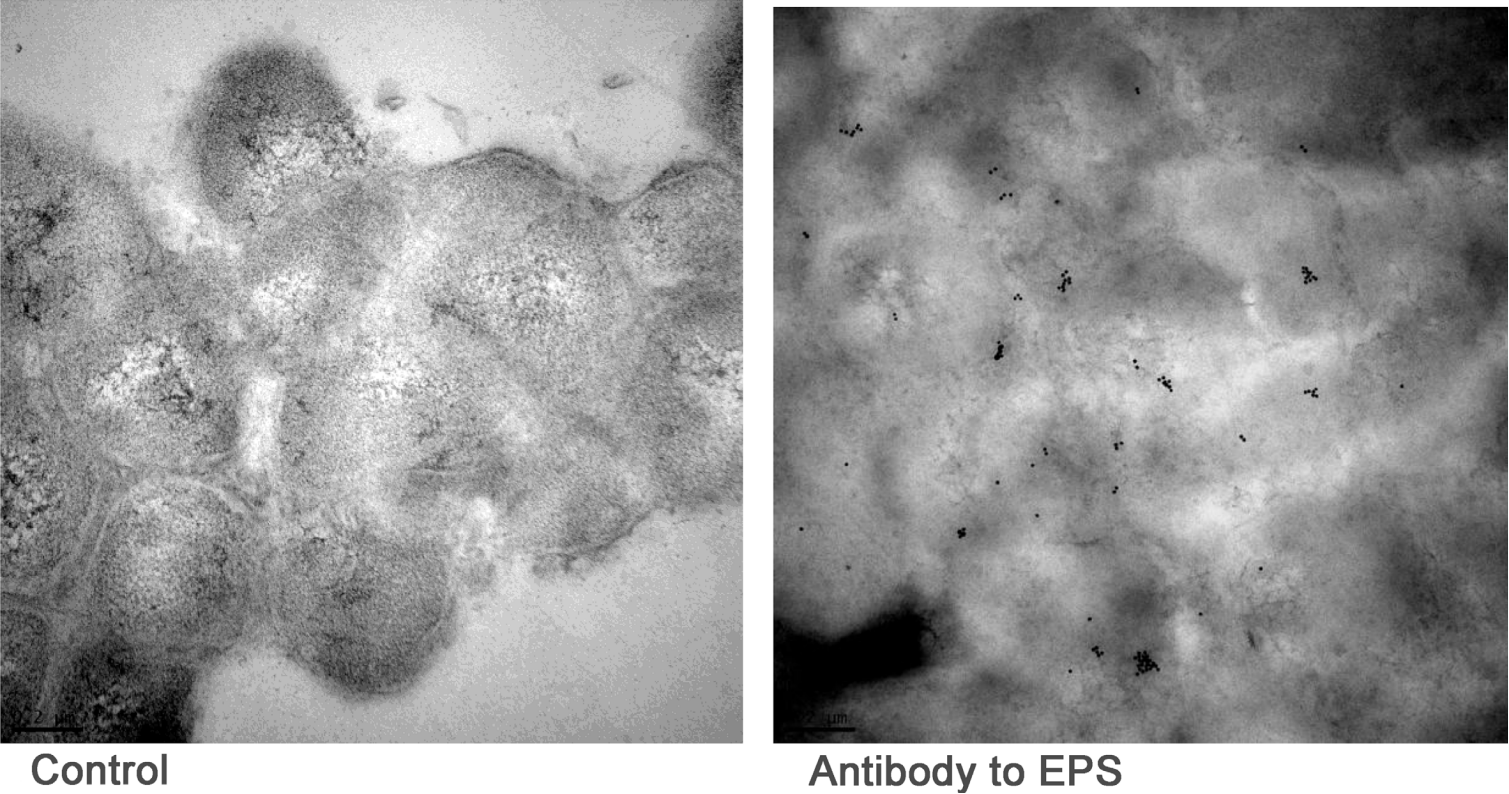
**Immuno-transmission electron micrographs of the OCT cryosection of an *H. somni *biofilm.***H. somni *was grown as a biofilm on glass slides and embedded in OCT resin to maintain the integrity of the biofilm prior to incubation with antiserum. Left, control OCT cryosection of biofilm incubated without specific antiserum, but with anti-rabbit conjugated gold particles; no labeling with the gold particles occurred; Right, OCT cryosection of a biofilm incubated with rabbit antibodies to EPS, followed by anti-rabbit conjugated gold particles. The black dots are gold particles around the bacterial cells and in the residual biofilm matrix.

The presence of EPS in the *H. somni *biofilm was examined by cryo-ITEM following incubation of the fixed samples with antiserum to EPS and Protein-A gold particles. The Protein-A gold particles bound to the bacterial surface and in spaces between the cells, which appeared to be the residual biofilm matrix. However, no gold particles were seen in the control sample incubated without antiserum (Figure 8).

**Figure 9:**
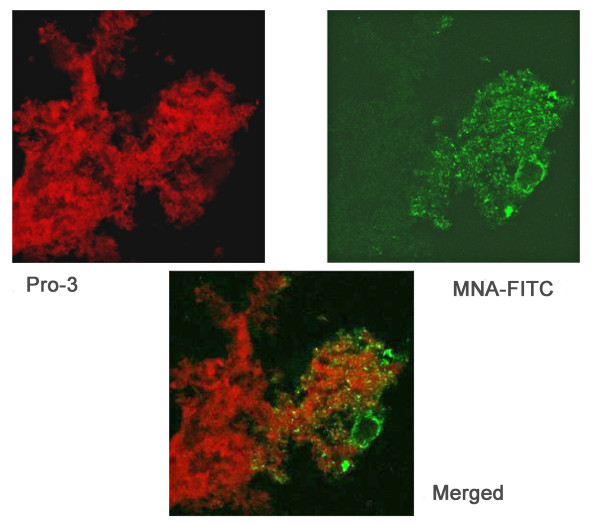
***H. somni *biofilm labeled with *Moringa *M lectin**. *H. somni *was grown as a biofilm on cover slips and stained with TO-PRO-3 to label the bacterial cells (top left), MNA (specific for α-mannose)-FITC to label mannose (top right), and were merged (bottom center) to demonstrate the presence of mannose within the bacterial biofilm. Mannose is present in the *H. somni *EPS, but not in the LOS.

**Figure 10:**
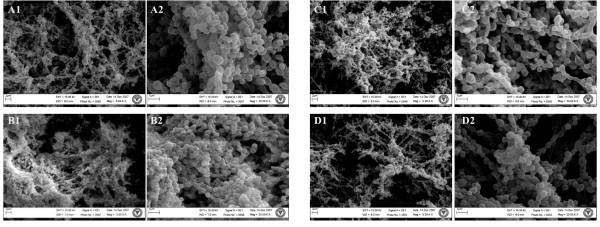
**SEM image of biofilm formation by *H. somni *2336 and 129Pt.** A1-A2, biofilm formation by 2336; B1- B2, enhanced biofilm formation by 2336 grown in the presence of Neu5Ac (50 μg/ml) in chemically defined medium; C1- C2, biofilm formation by 129Pt; D1- D2, biofilm formation by 129Pt grown in the presence of Neu5Ac in defined medium. There is no significant change in the density of the biofilm of 129Pt grown in the presence of Neu5Ac.

Mannose is not present in the *H. somni *LOS, but is the predominant component of the EPS. Therefore, a fluorescein isothionate-labeled, mannose-specific lectin (*Morniga M *[black mulberry]) was incubated with *H. somni *biofilms. This lectin bound to the matrix material between the cells of the biofilm of 2336 (Figure 9), indicating that the EPS was a major component of the *H. somni *biofilm. Analysis of the biofilm embedded in OCT resin with the sialic acid-reactive lectins (MAA [*Maackia amurensis*], WGA [Wheat Germ agglutinin], HHA [Amaryllis], and SBA [soybean] further supported that Neu5Ac was also a component of the biofilm of 2336 (data not shown). SEM examination showed that the addition of Neu5Ac to chemically defined medium increased biofilm production by 2336, whereas biofilm formation by 129Pt was unchanged (Figure 10). Although the LOS of 2336 was sialylated when grown in the presence of Neu5Ac, there were no differences in LOS structure or sialylation levels when 2336 was grown as a biofilm, as planktonic cells, or on blood agar plates (additional file 1, Table S1). In the absence of supplemental Neu5Ac, only LOS from 2336 grown on blood agar plates was sialylated, presumably due to the presence of Neu5Ac in the fresh blood. As previously reported [12], the LOS of 129Pt grown under any of the above conditions was not sialylated.

### Putative polysaccharide locus in *H. somni *2336

To understand the genetic basis of EPS biosynthesis in *H. somni*, we sought to identify a locus of genes that could encode for enzymes involved in the synthesis and transport of a polysaccharide other than LOS. A homolog of *manB*, which encodes for phosphomannomutase, was identified in the genomes of 2336 and 129Pt [2,25]. Phosphomannomutase is responsible for conversion of mannose-6-phosphate to mannose-1-phosphate. Furthermore, *manB *is flanked by *galU*, a glucose pyrophosphorylase, and *csrA*, a putative carbon storage regulator (Table 3 and additional file 2, Figure S1). Genome annotation also identified the presence of a ~19 kb region that contains a cluster of genes predicted to encode for glycosyltransferases, transport proteins, and other proteins involved in polysaccharide biosynthesis (Table 3 and additional file 2, Figure S1). The G+C content (36%) of this locus was similar to that of *H. somni *genomes (37%) [2,25].

**Table 3:** Putative EPS genes in *H.somni *2336 and 129Pt with proposed roles in polysaccharide synthesis

Gene	ORF (HSM-*H. somni *2336 and HS- *H. somni *129Pt)	Protein annotation	No. of amino acids, predicted mass (kDa)	% Similarity to another protein
*galU*	HSM_1063 HS_1117	UTP-glucose-1-phosphate uridylyltransferase	295, 32.2	70, to glucose-1-phosphate uridylyltransferase, *galU *(*E. coli*)
*manB*	HSM_1062 HS_1118	Phosphomannomutase	454, 50.3	81, to phosphomannomutase, *cpsG *(*E. coli*)
*csrA*	HSM_1061 HS_1119	Carbon storage regulator	60, 6.75	89, to pleiotropic regulatory protein for carbon source metabolism, *csrA *(*E. coli*)
*pldB*	HSM_1242 HS_0775	Lysophospholipase	318, 37.4	49, to lysophospholipase L2, *pldB *(*E. coli*)
*ybhA*	HSM_1241 HS_0774	Haloacid dehalogenase-like hydrolase	273, 30.8	60, to phosphatase//phospho transferase, *ybhA *(*E. coli*)
*araD*	HSM_1240 HS_0773	L-ribulose-5-phosphate 4-epimerase	231, 25.8	82, to L-ribulose-5-phosphate 4-epimerase, *yiaS *(*E. coli*)
*sgbU*	HSM_1239 HS_0772	Putative L-xylulose-5-phosphate 3-epimerase	290, 33.2	84, to L-xylulose 5-phosphate 3-epimerase, *yiaQ *(*E. coli*)
*rmpA*	HSM_1238 HS_0771	3-keto-L-gulonate-6-phosphate decarboxylase	215, 23.6	64, to 3-keto-L-gulonate 6-phosphate decarboxylase, *yiaQ *(*E. coli*)
*xylB*	HSM_1237 HS_0770	L-xylulose kinase	484, 53.7	75, to L-xylulose kinase, *lyxK *(*E. coli*)
*rbs1C*	HSM_1236 HS_0769	Ribose ABC transporter, permease	342, 32.9	59, to D-ribose transporter subunit, *rbsc *(*E. coli*)
*rbs1A*	HSM_1235 HS_0768	Ribose ABC transporter, ATPase component	496, 56.1	60, to D-ribose transporter subunit, ATP-binding component, *rbsA *(*E. coli *K12)
*rbs1B*	HSM_1234 HS_0767	ABC-type sugar transport system, periplasmic component	312, 31.0	56, to D-ribose transporter subunit, periplasmic component (*E. coli *)
*glsS*	HSM_1233 HS_0766	Gluconolaconase	295, 32.6	46, to gluconolactonase, *gnl* (*Zymomonas mobilis*)
*rbs2B*	HSM_1232 HS_0765	ABC-type sugar-binding periplasmic protein	369, 37.2	81, to hypothetical protein (*Yersinia intermedia *ATCC 29909)
*rbs2C*	HSM_1231 HS_0764	Ribose ABC transporter, permease	349, 36.9	90, to inner-membrane translocator (*Yersinia intermedia *ATCC 29909)
*rbs2A*	HSM_1230 HS_0763	Ribose ABC transporter, ATPase component	505, 55.8	81, to ABC transporter-related protein (*Yersinia intermedia *ATCC 29909)
*dctP*	HSM_1229 HS_0762	TRAP C4-dicarboxylate transport system, periplasmic component	328, 33.4	52, to C4-dicarboxylate binding protein, periplasmic component, *dctP *(*Rhodobacter capsulatus*)
*dctM*	HSM_1228 HS_0761	TRAP C4-dicarboxylate transport system, permease component	426, 43.2	59, to C4-dicarboxylate -binding protein, permease component, *dctM (Rhodobacter capsulatus*)
*dctQ*	HSM_1227 HS_0760	Tripartite ATP-independent periplasmic transporter	160, 17.8	40, to tripartite ATP-independent periplasmic transporter, *dctQ* (*Rhodobacter capsulatus*)

### Differential gene expression in biofilm and planktonic cells

**Figure 11:**
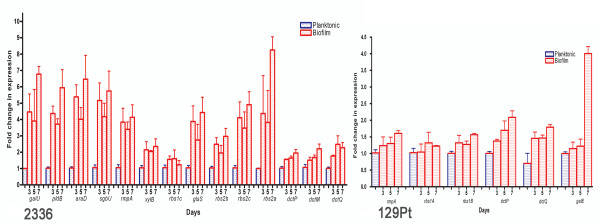
**Genes predicted to contribute to EPS biosynthesis that were significantly (P < 0.05) upregulated during biofilm growth (red bars) relative to planktonic growth (blue bars)**. The bacteria were grown as biofilms or in broth (planktonic) and samples taken at 3, 5 and 7 days for analysis by qRT-PCR from *H. somni *2336 (left) or 129Pt (right). Fourteen of 19 genes were significantly upregulated in 2336, whereas only 5 genes were upregulated (predominately at 7 days) in 129Pt. The data were expressed as the means and SDs of three independent experiments performed in triplicate.

Among the 19 genes in the two loci described above, fourteen genes were upregulated when *H. somni *2336 was grown under conditions favorable to biofilm formation, compared to planktonic-grown cells (Figure 11). The greatest level of induction (8-fold) when the cells were in biofilm phase occurred for *rbs2a*, which had the greatest sequence similarity to a gene encoding for an ATP-binding constituent of the ribose ATP-binding cassette protein (ABC) transporter. Furthermore, *rbs2b *and *rbs2c*, which are similar to genes encoding for a periplasmic substrate-binding protein and a transmembrane constituent of the ribose ABC transporter, respectively, were also upregulated in biofilm phase cells (Table 3). *H. somni galU*, which is essential for galactose utilization and synthesis of a variety of carbohydrates, was upregulated 7-fold when grown in the biofilm phase compared to planktonic growth, supporting the potential role of this gene for EPS biosynthesis (Figure 11). The putative functions of other genes, which were upregulated 2-5 fold (Figure 11) are described in Table 3. In contrast to the large number of genes in this locus that were upregulated when 2336 was grown as a biofilm, only five genes in this locus were upregulated, and then only 1-2 fold, when 129Pt was grown as a biofilm (Figure 11). These results supported that these loci contributed to EPS production, and were consistent with previous results that the biofilm is thicker and larger in 2336 compared to 129Pt [29]. In addition to the genes in the putative EPS loci, expression of *siaB*, which encodes for alpha-2,3-sialyltransferase, was upregulated 15-fold when 2336 was grown as a biofilm compared to planktonic cells (data not shown).

## Discussion

It is now established that *H. somni *makes an excellent biofilm *in vitro *and *in vivo *[29,49], but the nature of the biofilm matrix has not been characterized. The biofilm matrix of most bacterial cells contains polysaccharide that is upregulated under conditions that favor biofilm growth, such as the EPS in the biofilm of *Pseudomonas aeruginosa *[50,51]. Miller et al. [28] reported the presence of a polysaccharide in the supernatant of *H. somni *colonies washed off culture plates. However, the nature and composition of this polysaccharide was not reported, and it was not differentiated from LOS.

Anaerobiosis is also commonly associated with host infections and the substratum of biofilms [52]. Therefore, we sought to determine if the phenotype of *H. somni *changed when the bacteria were grown under anaerobic conditions. Although there was no substantial change in the LOS profile or outer membrane protein profile, which occurs when *N. gonorrhoeae *is grown anaerobically [53], a high molecular size polysaccharide was produced by *H. somni *under anaerobic growth conditions. Furthermore, production of this polysaccharide was enhanced under other stress conditions, such as stationary phase, increased salt content, and conditions that favor biofilm formation. Therefore, this polysaccharide is likely to be produced in the host, where the competition for nutrients and the host response continually stresses bacterial cells. The polysaccharide did not appear to be attached to the cell surface, and was therefore consistent with it being an EPS rather than a capsule. The failure to previously characterize this EPS was due to the fact that little, if any, of this material was produced during log phase (planktonic growth) in broth.

Purification of the EPS was initially difficult due to poor growth of the bacteria under anaerobic conditions and the relatively small amount of EPS made even in stationary phase broth cultures. The greatest amount of EPS:cell mass ratio was clearly produced under conditions that favored biofilm formation. The chemical structure of the EPS from 2336 was that of a complex, branched, galacto-mannan polymer consisting of a 6-substituted mannose framework that branched at C-2 with occasional galactose residues at the non-reducing end of the tetrasaccharide branch. This structure is remarkably similar to that of yeast mannan [54]. Attempts to purify a mannan-containing polysaccharide from the growth medium alone, including supplemented BHI, Terrific broth, and Columbia broth, were unsuccessful, confirming that this material was not derived from yeast extract in the medium. Antibodies to the EPS and the lectin *Morniga M *(MNA; specific for α-mannose, which is only present in the EPS) bound to and between *H. somni *cells grown in a biofilm, indicating the EPS was part of the biofilm matrix.

Due to the presence of terminal galactose residues in the EPS, and that *H. somni *can sialylate the terminal galactose residues of its LOS, we sought to determine if the EPS could also be sialylated. GC-MS confirmed that when 2336 was grown in the presence of Neu5Ac the EPS was sialylated, but the EPS of 129Pt was not. The lack of sialylation of 129Pt EPS was expected as this strain lacks the sialyltransferases and Neu5Ac-synthetase required to attach Neu5Ac to galactose residues [25]. However, there was no difference in the sialylation of LOS glycoforms in planktonic, plate-grown, or biofilm-grown cells, suggesting that Neu5Ac promoted biofilm formation in *H. somni *2336 through sialylation of the EPS.

In *H. somni *the presence of Neu5Ac on the LOS reduces antibody binding and promotes serum resistance [12,55]. Neu5Ac is also a normal component of host cells, thereby mimicking human oligosaccharides [7]. Neu5Ac on the LOS also binds to complement factor H [56], and protects the bacteria from complement-mediated killing [57]. In nontypable *H. influenzae *(NTHI), which does not produce a known EPS, sialylation of the LOS promotes biofilm formation [58]. Neu5Ac is a terminal sugar of the NTHI biofilm matrix [59] and is required for biofilm formation in the otitis media Chinchilla model [60]. Inactivation of *siaB *(CMP-Neu5Ac synthetase) prevents addition of Neu5Ac onto the LOS and attenuates the mutant in the otitis media model, in which biofilm is a predominant component [60,61].

A BLAST search of the genome sequences of 2336 and 129Pt identified putative genes in two regions that could encode for proteins responsible for EPS synthesis [[25], Siddaramappa S CJ, Duncan AJ, Gillaspy AF, Carson M, Gipson J, Gipson M, Orvis J, Zaitshik J, Barnes G, Brettin TS, Bruce D, Chertkov O, Detter JC, Han CS, Tapia R, Thompson LS, Dyer DW, Inzana TJ: Genome sequence of *Histophilus somni *strain 2336 from bovine pneumonia and comparison to commensal strain 129Pt reveals extensive horizontal gene transfer and evolution of pathogenesis. Submitted]. One locus contained 16 genes with similarity to genes responsible for carbohydrate assembly, transport, and polysaccharide synthesis. Another region contained genes with high homology to *galU, manB*, and *csr*, which could be involved in the synthesis of any polymer containing galactose and mannose. The putative functions of the products of some of these genes resemble those of the *P. aeruginosa psl *(polysaccharide synthesis locus), which consists of a group of 15 genes encoding for enzymes responsible for synthesis of the mannose- and galactose-rich biofilm-associated EPS [50,62,63]. Attempts to mutate any of these *H. somni *genes by allelic replacement using pGEM3Z, as previously described [10], or other *H. somni *suicide vectors were unsuccessful. Therefore, qRT-PCR was used to determine if enhanced expression of the EPS, which occurs during biofilm formation, correlated with upregulation of the putative EPS locus. More than two-thirds of the genes in this locus were significantly upregulated when the bacteria were grown under conditions favorable to biofilm formation (and EPS production), compared to planktonic growth. Some of the putative genes with the largest induction in expression were those encoding for periplasmic substrate-binding proteins and transmembrane constituents of the ribose ABC transporter. This family of ABC transporters represents domain II of the carbohydrate uptake proteins that transport only monosaccharides. In *E. coli*, mutations in any of these genes (*rbsa, rbsb, rbsc*) eliminates transport of ribose, indicating that these components form a transport system that is responsible for high-affinity ribose transport [64]. The gene *galU*, which encodes for glucose-1-phosphate uridylyltransferase, was also highly upregulated and is responsible for catalyzing the reversible production of UDP-glucose. The gene *galU *plays a pivotal role in the synthesis of the carbohydrate moieties of glycolipids, glycoproteins, and proteoglycans. *galU *is also essential for capsular polysaccharide biosynthesis in *Streptococcus pneumoniae *[65]. In *H. influenzae, galU *is an essential housekeeping gene that is important in generating sugar precursors needed for polysaccharide formation and LOS outer core synthesis [66]. The *H. somni *GalU in this locus is 70% similar to that of *H. influenzae *at the amino acid level. Of interest was that in 129Pt only 5 of the genes in these two loci were significantly upregulated when the bacteria were grown under conditions favorable to biofilm formation, which is much thinner and less substantial than that of 2336 [29], and much less EPS can be isolated from the biofilm of 129Pt (data not shown). Therefore, these experiments support the premise that these genes encode for proteins responsible for EPS biosynthesis.

It will be important to determine if all or most strains of *H. somni *produce an antigenically identical or similar EPS, and if antibodies to the EPS can be used to differentiate infected animals from healthy, colonized animals. Preliminary ELISA experiments with antibodies to the EPS indicated that most strains do produce this EPS. Serological studies with infected and healthy animals are in progress.

## Conclusions

We describe the isolation and structure of an *H. somni *EPS. The EPS was upregulated under stress-like conditions, and appeared to be a major component of the matrix of the *H. somni *biofilm. An attractive hypothesis is that formation of EPS and a biofilm is, in part, responsible for the capability of *H. somni *to persist in tissues and cause chronic infections. Since biofilm formation in the bovine host occurs during disease [49], it will be important to determine if compounds that inhibit EPS production will reduce biofilm formation in the host and hasten recovery. The putative genes responsible for EPS synthesis were also identified, which will lead to the development of mutants unable to synthesize EPS and determine the role of the EPS and biofilm in virulence. Furthermore, if EPS is produced primarily during the disease process, this antigen may prove useful in serological assays for diagnosis of *H. somni *infection.

## List of abbreviations

EPS: exopolysaccharide; Neu5Ac: *N*-acetylneuraminic acid; qRT-PCR: quantitative real-time reverse transcription-polymerase chain reaction; LOS: lipooligosaccharide; CBA: Columbia agar with 5% sheep blood; CTT: Columbia broth supplemented with 0.1% Trizma base and 0.01% thiamine monophosphate; TTT: Terrific broth supplemented with 0.1% Trizma base and 0.01% thiamine monophosphate; TMP: thiamine monophosphate; CFU, colony forming units; PBS: phosphate buffered saline, pH 7.2; Cetavlon: hexadecyltrimethyl ammonium bromide; PAGE: polyacrylamide gel electrophoresis; ITEM: immuno-transmission electron microscopy; NGS: normal goat serum; SEM: scanning electron microscopy; OdA: O-deacylated; ES-MS: electrospray mass spectrometry; GLC-MS: gas-liquid chromatography-mass spectrometry; NMR: nuclear magnetic resonance spectroscopy; ROESY: rotating frame Overhauser enhancement spectroscopy; COSY: correlation spectroscopy; TOCSY: total correlation spectroscopy experiments; DQF-COSY: double quantum filtered correlation spectroscopy; HMBC: heteronuclear multiple bond correlation; HSQC: heteronuclear single quantum coherence; C_t_: critical threshold cycle; ABC: ATP-binding cassette; NTHI: nontypable *H. influenzae*.

## Authors' contributions

IS carried out the scanning qRT-PCR, electron microscopy, and biofilm studies, TJI was responsible for the identification and purification of the EPS and electrophoretic techniques, MAA and JQS carried out the freeze-fracture ITEM and lectin binding studies, AM and CDC carried out analytical and structural analyses of the EPS, ADC and FAM carried out analytical studies on the EPS and LOS, GB carried out preparation of the immune sera, ITEM of EPS on whole cells, and electrophoretic methods. IS, TJI, and AM wrote the manuscript. All authors read and approved the final manuscript.

## Supplementary Material

Additional file 1 **Proposed composition of OdA LOS from *H. somni *strains 2336 and 129Pt grown as a biofilm, as planktonic cells, or on blood agar plates by negative-ion-ES-MS**. Data of the observed ions (*m/z*), observed and calculated molecular mass (in daltons), and proposed composition of O-deacylated lipooligosaccharides from *H. somni *strains 2336, which can be sialylated, and 129Pt, which is cannot be sialylated, grown with and without sialic acid as a biofilm, planktonically, and on blood agar. Click here for file

Additional file 2 **Maps of *H. somni *2336 chromosomal loci containing genes proposed to encode for proteins involved in EPS biosynthesis**. A, an ~19 kb region containing genes predicted to encode for glycosyltransferases and transport proteins; B, an ~3 kb region that contains *manB*. For detailed analyses of the putative gene products see Table 3. Click here for file
