# Supplementary material for: Identification, structure, and characterization of an exopolysaccharide produced by Histophilus somni during biofilm formation
Source: BMC Microbiol. 2011 Aug 19;11:186. doi: 10.1186/1471-2180-11-186 (PMC3224263; doi:10.1186/1471-2180-11-186)
Supplement: Additional file 1 — Proposed composition of OdA LOS from H. somni strains 2336 and 129Pt grown as a biofilm, as planktonic cells, or on blood agar plates by negative-ion-ES-MS. Data of the observed ions (m/z), observed and calculated molecular mass (in daltons), and proposed composition of O-deacylated lipooligosaccharides from H. somni strains 2336, which can be sialylated, and 129Pt, which is cannot be sialylated, grown with and without sialic acid as a biofilm, planktonically, and on blood agar. [file 1471-2180-11-186-S1.DOCX]

**Additional Table 1.** Proposed composition of OdA LOS from *H. somni* strains 2336 and 129Pt grown as a biofilm, as planktonic cells, or on blood agar plates by negative-ion-ES-MS

| **Strain** | **Observed Ions (m*/z*)**  **(M-2H)^2-^ (M-H)^3-^** | | **Molecular Mass (Da)**  **Observed Calculated** | | **Proposed Composition** |
| --- | --- | --- | --- | --- | --- |
| 2336 grown as a biofilm  without Neu5Ac | 1112.0 | 741.0 | 2226.0 | 2224.1 | 2Hex, 2Hep, PEtn, 2Kdo, Lipid A-OH |
|  | - | 782.0 | 2349.0 | 2347.1 | 2Hex, 2Hep, 2PEtn, 2Kdo, Lipid A-OH |
|  | 1193.0 | 795.0 | 2388.0 | 2386.3 | 3Hex, 2Hep, PEtn, 2Kdo, Lipid A-OH |
|  | 1254.5 | 836.0 | 2509.5 | 2509.3 | 3Hex, 2Hep, 2PEtn, 2Kdo, Lipid A-OH |
|  | - | 862.5 | 2590.5 | 2589.5 | 3Hex, HexNAc, 2Hep, PEtn, 2Kdo, Lipid A-OH |
|  |  | 916.2 | 2751.6 | 2751.6 | 4Hex, HexNAc, 2Hep, PEtn, 2Kdo, Lipid A-OH |
|  |  | 958.0 | 2877.0 | 2874.7 | 4Hex, HexNAc, 2Hep, 2PEtn, 2Kdo, Lipid A-OH |
| 2336 grown as a biofilm  with Neu5Ac | 1112.0 | 741.0 | 2226.0 | 2224.1 | 2Hex, 2Hep, PEtn, 2Kdo, Lipid A-OH |
|  | - | 782.0 | 2349.0 | 2347.1 | 2Hex, 2Hep, 2PEtn, 2Kdo, Lipid A-OH |
|  | 1193.0 | 795.0 | 2388.0 | 2386.3 | 3Hex, 2Hep, PEtn, 2Kdo, Lipid A-OH |
|  | 1254.5 | 836.0 | 2509.5 | 2509.3 | 3Hex, 2Hep, 2PEtn, 2Kdo, Lipid A-OH |
|  | - | 862.5 | 2590.5 | 2589.5 | 3Hex, HexNAc, 2Hep, PEtn, 2Kdo, Lipid A-OH |
|  | - | 892.0 | 2679.0 | 2678.5 | Sial, 3Hex, 2Hep, PEtn, 2Kdo, Lipid A-OH |
|  |  | 916.2 | 2751.6 | 2751.6 | 4Hex, HexNAc, 2Hep, PEtn, 2Kdo, Lipid A-OH |
|  | - | 933.0 | 2802.0 | 2801.6 | Sial, 3Hex, 2Hep, 2PEtn, 2Kdo, Lipid A-OH |
|  |  | 958.0 | 2877.0 | 2874.7 | 4Hex, HexNAc, 2Hep, 2PEtn, 2Kdo, Lipid A-OH |
|  | - | 1014.0 | 3045.0 | 3043.8 | Sial, HexNAc, 4Hex, 2Hep, PEtn, 2Kdo, Lipid A-OH |
| 2336 grown planktonic-ally without Neu5Ac | - | - | - | - | No spectrum could be obtained from this sample |
| 2336 grown planktonic-ally with Neu5Ac | 1112.0 | 741.0 | 2226.0 | 2224.1 | 2Hex, 2Hep, PEtn, 2Kdo, Lipid A-OH |
|  | - | 782.0 | 2349.0 | 2347.1 | 2Hex, 2Hep, 2PEtn, 2Kdo, Lipid A-OH |
|  | 1193.0 | 795.0 | 2388.0 | 2386.3 | 3Hex, 2Hep, PEtn, 2Kdo, Lipid A-OH |
|  | 1254.5 | 836.0 | 2509.5 | 2509.3 | 3Hex, 2Hep, 2PEtn, 2Kdo, Lipid A-OH |
|  | - | 862.5 | 2590.5 | 2589.5 | 3Hex, HexNAc, 2Hep, PEtn, 2Kdo, Lipid A-OH |
|  | - | 892.0 | 2679.0 | 2678.5 | Sial, 3Hex, 2Hep, PEtn, 2Kdo, Lipid A-OH |
|  |  | 916.2 | 2751.6 | 2751.6 | 4Hex, HexNAc, 2Hep, PEtn, 2Kdo, Lipid A-OH |
|  | - | 933.0 | 2802.0 | 2801.6 | Sial, 3Hex, 2Hep, 2PEtn, 2Kdo, Lipid A-OH |
|  |  | 958.0 | 2877.0 | 2874.7 | 4Hex, HexNAc, 2Hep, 2PEtn, 2Kdo, Lipid A-OH |
|  | - | 1014.0 | 3045.0 | 3043.8 | Sial, HexNAc, 4Hex, 2Hep, PEtn, 2Kdo, Lipid A-OH |
|  |  | 1055.0 | 3168.0 | 3166.9 | Sial, HexNAc, 4Hex, 2Hep, 2PEtn, 2Kdo, Lipid A-OH |
| 2336 grown on blood agar without Neu5Ac | 1112.0 | 741.0 | 2226.0 | 2224.1 | 2Hex, 2Hep, PEtn, 2Kdo, Lipid A-OH |
|  | - | 782.0 | 2349.0 | 2347.1 | 2Hex, 2Hep, 2PEtn, 2Kdo, Lipid A-OH |
|  | 1193.0 | 795.0 | 2388.0 | 2386.3 | 3Hex, 2Hep, PEtn, 2Kdo, Lipid A-OH |
|  | 1254.5 | 836.0 | 2509.5 | 2509.3 | 3Hex, 2Hep, 2PEtn, 2Kdo, Lipid A-OH |
|  | - | 862.5 | 2590.5 | 2589.5 | 3Hex, HexNAc, 2Hep, PEtn, 2Kdo, Lipid A-OH |
|  |  | 916.2 | 2751.6 | 2751.6 | 4Hex, HexNAc, 2Hep, PEtn, 2Kdo, Lipid A-OH |
|  |  | 958.0 | 2877.0 | 2874.7 | 4Hex, HexNAc, 2Hep, 2PEtn, 2Kdo, Lipid A-OH |
| 2336 grown on blood agar with Neu5Ac | 1112.0 | 741.0 | 2226.0 | 2224.1 | 2Hex, 2Hep, PEtn, 2Kdo, Lipid A-OH |
|  | - | 782.0 | 2349.0 | 2347.1 | 2Hex, 2Hep, 2PEtn, 2Kdo, Lipid A-OH |
|  | 1193.0 | 795.0 | 2388.0 | 2386.3 | 3Hex, 2Hep, PEtn, 2Kdo, Lipid A-OH |
|  | 1254.5 | 836.0 | 2509.5 | 2509.3 | 3Hex, 2Hep, 2PEtn, 2Kdo, Lipid A-OH |
|  | - | 862.5 | 2590.5 | 2589.5 | 3Hex, HexNAc, 2Hep, PEtn, 2Kdo, Lipid A-OH |
|  | - | 892.0 | 2679.0 | 2678.5 | Sial, 3Hex, 2Hep, PEtn, 2Kdo, Lipid A-OH |
|  |  | 916.2 | 2751.6 | 2751.6 | 4Hex, HexNAc, 2Hep, PEtn, 2Kdo, Lipid A-OH |
|  | - | 933.0 | 2802.0 | 2801.6 | Sial, 3Hex, 2Hep, 2PEtn, 2Kdo, Lipid A-OH |
|  |  | 958.0 | 2877.0 | 2874.7 | 4Hex, HexNAc, 2Hep, 2PEtn, 2Kdo, Lipid A-OH |
|  | - | 1014.0 | 3045.0 | 3043.8 | Sial, HexNAc, 4Hex, 2Hep, PEtn, 2Kdo, Lipid A-OH |
| 129Pt grown as a biofilm without Neu5Ac | 1132.5 | 754.5 | 2266.8 | 2265.1 | Hex, HexNAc, 2Hep, PEtn, 2Kdo, Lipid A-OH |
|  | 1212.8 | 808.5 | 2428.0 | 2427.2 | 2Hex, HexNAc, 2Hep, PEtn, 2Kdo, Lipid A-OH |
|  | 1294.5 | 862.5 | 2590.7 | 2589.4 | 3Hex, HexNAc, 2Hep, PEtn, 2Kdo, Lipid A-OH |
| 129Pt grown as a biofilm with Neu5Ac | 1132.5 | 754.5 | 2266.8 | 2265.1 | Hex, HexNAc, 2Hep, PEtn, 2Kdo, Lipid A-OH |
|  | 1212.8 | 808.5 | 2428.0 | 2427.2 | 2Hex, HexNAc, 2Hep, PEtn, 2Kdo, Lipid A-OH |
|  | 1294.5 | 862.5 | 2590.7 | 2589.4 | 3Hex, HexNAc, 2Hep, PEtn, 2Kdo, Lipid A-OH |
| 129Pt grown planktonic-ally without Neu5Ac | 1132.5 | 754.5 | 2266.8 | 2265.1 | Hex, HexNAc, 2Hep, PEtn, 2Kdo, Lipid A-OH |
|  | 1212.8 | 808.5 | 2428.0 | 2427.2 | 2Hex, HexNAc, 2Hep, PEtn, 2Kdo, Lipid A-OH |
|  | 1294.5 | 862.5 | 2590.7 | 2589.4 | 3Hex, HexNAc, 2Hep, PEtn, 2Kdo, Lipid A-OH |
| 129Pt grown planktonic-ally with Neu5Ac | 1132.5 | 754.5 | 2266.8 | 2265.1 | Hex, HexNAc, 2Hep, PEtn, 2Kdo, Lipid A-OH |
|  | 1212.8 | 808.5 | 2428.0 | 2427.2 | 2Hex, HexNAc, 2Hep, PEtn, 2Kdo, Lipid A-OH |
|  | 1294.5 | 862.5 | 2590.7 | 2589.4 | 3Hex, HexNAc, 2Hep, PEtn, 2Kdo, Lipid A-OH |
| 129Pt grown on blood agar without Neu5Ac | 1132.5 | 754.5 | 2266.8 | 2265.1 | Hex, HexNAc, 2Hep, PEtn, 2Kdo, Lipid A-OH |
|  | 1212.8 | 808.5 | 2428.0 | 2427.2 | 2Hex, HexNAc, 2Hep, PEtn, 2Kdo, Lipid A-OH |
|  | 1294.5 | 862.5 | 2590.7 | 2589.4 | 3Hex, HexNAc, 2Hep, PEtn, 2Kdo, Lipid A-OH |
| 129Pt grown on blood agar with Neu5Ac | 1132.5 | 754.5 | 2266.8 | 2265.1 | Hex, HexNAc, 2Hep, PEtn, 2Kdo, Lipid A-OH |
|  | 1212.8 | 808.5 | 2428.0 | 2427.2 | 2Hex, HexNAc, 2Hep, PEtn, 2Kdo, Lipid A-OH |
|  | 1294.5 | 862.5 | 2590.7 | 2589.4 | 3Hex, HexNAc, 2Hep, PEtn, 2Kdo, Lipid A-OH |
