# Supplementary figures and images for: Identification, structure, and characterization of an exopolysaccharide produced by Histophilus somni during biofilm formation
Source: BMC Microbiol. 2011 Aug 19;11:186. doi: 10.1186/1471-2180-11-186 (PMC3224263; doi:10.1186/1471-2180-11-186)

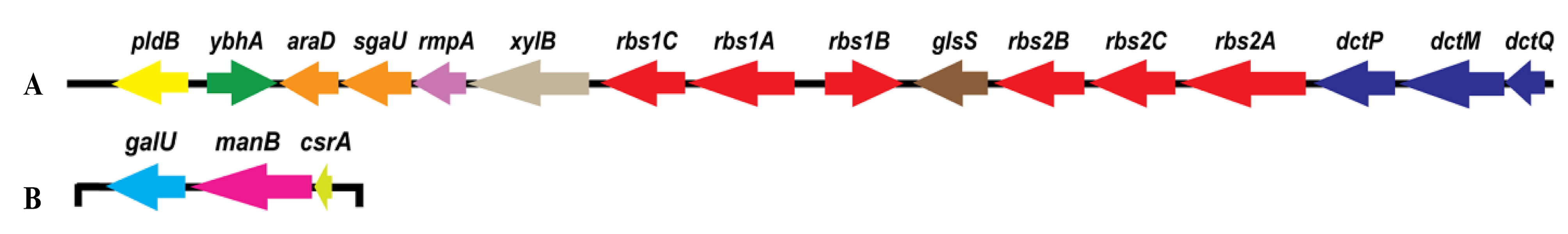

Supplement: Additional file 2 — Maps of H. somni 2336 chromosomal loci containing genes proposed to encode for proteins involved in EPS biosynthesis. A, an ~19 kb region containing genes predicted to encode for glycosyltransferases and transport proteins; B, an ~3 kb region that contains manB. For detailed analyses of the putative gene products see Table 3. [file 1471-2180-11-186-S2.TIFF]
